# Linking Autophagy to Potential Agronomic Trait Improvement in Crops

**DOI:** 10.3390/ijms23094793

**Published:** 2022-04-26

**Authors:** Jingran Wang, Shulei Miao, Yule Liu, Yan Wang

**Affiliations:** MOE Key Laboratory of Bioinformatics, Center for Plant Biology, School of Life Science, Tsinghua University, Beijing 100084, China; wangjr19@mails.tsinghua.edu.cn (J.W.); miao1094284714@163.com (S.M.); yuleliu@mail.tsinghua.edu.cn (Y.L.)

**Keywords:** autophagy, agronomic trait, nutrient recycling and remobilization, yield, abiotic stress, biotic stress, autophagy manipulation, crops

## Abstract

Autophagy is an evolutionarily conserved catabolic process in eukaryotic cells, by which the superfluous or damaged cytoplasmic components can be delivered into vacuoles or lysosomes for degradation and recycling. Two decades of autophagy research in plants uncovers the important roles of autophagy during diverse biological processes, including development, metabolism, and various stress responses. Additionally, molecular machineries contributing to plant autophagy onset and regulation have also gradually come into people’s sights. With the advancement of our knowledge of autophagy from model plants, autophagy research has expanded to include crops in recent years, for a better understanding of autophagy engagement in crop biology and its potentials in improving agricultural performance. In this review, we summarize the current research progress of autophagy in crops and discuss the autophagy-related approaches for potential agronomic trait improvement in crop plants.

## 1. Introduction

Unlike animals, plants can hardly escape from stressful environmental conditions unsuitable for growth and living, such as nutrient limitation, drought, waterlogging, salt, heat, cold, and pathogen infection. To survive, plants have evolved a series of mechanisms to adapt to these stresses, among which autophagy (meaning self-eating) is an important pathway for stress adaption. During autophagy, the unneeded or damaged intracellular macromolecules, such as proteins, lipids, nucleic acids, carbohydrate reserves, and organelles, can be delivered into vacuoles (yeasts and plants) or lysosomes (animals) for degradation and recycling [1,2]. In plants, two major autophagic pathways, macroautophagy and microautophagy, have been well-described and studied. Macroautophagy is characterized by the formation of a kind of double-membrane vesicle called an autophagosome, by which the cytoplasmic components are engulfed and transported to vacuoles for clearance, while microautophagy is achieved by the direct invagination of the tonoplast to capture cytoplasmic substrates for vacuolar breakdown [3]. Since macroautophagy is the best-elucidated type of autophagy, autophagy is hereafter used in the present study to indicate macroautophagy, unless otherwise specified in the review.

In the last two decades, research on plant autophagy has greatly advanced our understanding of the molecular mechanisms and physiological roles of autophagy, mainly based on studies conducted in model plants, such as *Arabidopsis thaliana* and *Nicotiana benthamiana*. Among the 20 autophagy-related (ATG) proteins established to be essential for autophagosome biogenesis in yeast [4], most of their counterparts in plants have been identified, except ATG17, ATG29, ATG31, and ATG38. These core ATG proteins in yeast can be classified into six groups, according to their functions: (1) the Atg1/ULK protein kinase complex (Atg1, Atg13, Atg11, Atg17, Atg29, and Atg31) contributing to the initiation of autophagosome biogenesis; (2) Atg9-containing vesicles involved in autophagosome precursor formation; (3) PI3K complex I (Vps15,Vps34, Atg6, Atg14, and Atg38) responsible for PtdIns3P generation; (4) Atg2–Atg18 complex acting as a tether between the autophagosome precursor and endoplasmic reticulum (ER) to mediate lipid transfer from ER; (5) the Atg8 lipidation system (Atg8, Atg4, Atg7, and Atg3) to promote autophagosome membrane expansion; and (6) the Atg12–Atg5–Atg16 complex (Atg12, Atg7, Atg10, Atg5, and Atg16) to stimulate the lipid conjugation reaction of Atg8 [4]. Albeit missing in plants and mammals, the roles of ATG17, ATG29, and ATG31 have been functionally substituted by ATG11/FIP200 and ATG101, while the issue concerning whether a counterpart of ATG38 exists in plants remains elusive. Therefore, autophagy is a highly conserved process in eukaryotes. Emerging evidence from reverse genetic studies in model plants indicates that autophagy participates in many important biological processes associated with agronomic traits, including nutrient remobilization, vegetative growth, reproduction, and stress tolerance [2,5,6,7,8,9]. Encouraged by those findings, autophagy research has rapidly extended to include crops. The genome-wide identification of *ATG* genes has been reported in multiple crop species, and functional studies revealing the significance of autophagy for crop productivity and quality have emerged in recent years. In this review, we mainly focus on the advances of autophagy research in crops, as well as the key findings of autophagy associated with important agronomic traits, and discuss the potential applications of autophagy for crop improvement. 

## 2. The Role of Autophagy in Nutrient Recycling and Remobilization 

### 2.1. The Acute Response of Autophagy to Nutrient Deprivation and Leaf Senescence

Plant growth and development are highly dependent on nutrient availability, and thus the ability of plants to recycle and remobilize nutrients is critical for their survival and productivity, especially during nutrient-limitation conditions. As the most well-known nutrient-starvation response in eukaryotes, autophagy can assist cells to cope with energy limitation by degrading intracellular substances for recycling and reuse. In plants, the importance of autophagy for survival under nutrient starvation has also been firstly indicated by the enhanced hypersensitivity of autophagy-defective plants to nitrogen deprivation or carbon starvation caused by sucrose-free growth medium or dark incubation. More severe symptoms of nutrient deficiency are observed in atg mutants, including reduced growth and accelerated leaf chlorosis, than the wild-type controls [10,11,12,13,14,15,16,17,18,19,20,21]. The upregulated expression of *ATG* genes and increased biogenesis of autophagic structures upon nitrogen or carbon starvation, indicated by the two well-established cytological markers, the fluorescent fusion proteins of ATG8 or the fluorescent dye monodansylcadaverine (MDC), further suggest the participation of autophagy in plant adaptation to nitrogen or carbon deficiency [11,19,20,22,23]. Recently, autophagy in *Arabidopsis* has been found to also be responsive to other nutrient-deficiency conditions, including zinc (Zn), sulfur (S), and inorganic phosphate (Pi) deprivation [22,23,24,25]. Autophagy-deficient *Arabidopsis* mutants show stunted growth and accelerated chlorosis under Zn limitation, and their growth recovery from Zn limitation is also limited [23,25]. S limitation causes earlier leaf-yellowing phenotype in the *Arabidopsis atg5* mutant [26], and P and Zn deficiency lead to an exacerbated inhibition of primary root growth in *atg* mutants [22,25]. All these findings in model plants demonstrate that autophagy is an important process for plant survival under nutrient limitation. 

For crops, the upregulated expression of *ATG* genes has also been validated in soybean, maize, rice, barley, pepper, foxtail millet, and apple exposed to nutrient stresses [13,27,28,29,30,31,32,33]. Additionally, nitrogen deprivation in maize plants enhances the total and lipidated ATG8 protein levels in the older leaves at 15–20 days after germination, and stimulates autophagosome biogenesis in the root cells, as indicated by the number of YFP-ZmATG8a-positive puncta [13,30]. Increased autophagic structures are also observed in maize-leaf protoplasts cultured in a sucrose-free medium [13]. However, when autophagy is deficient, exacerbated impacts of nutrient deficiency on growth are also observed in autophagy mutants of crop species, such as maize and rice. Maize *atg12* plants show dramatic reductions in root and shoot growth and enhanced leaf senescence under low nitrogen conditions, although their vegetative growth rates under high nitrogen conditions are similar to wild-type plants [13]. The rice autophagy-defective mutant, *Osatg7-1*, shows reduced growth compared to wild-type plants, even under normal conditions, and this growth difference is exacerbated further by nitrogen starvation [34]. Similarly, severe growth retardation is also observed in the sucrose-starved cultured cells of autophagy-defective rice *Osatg7-1* mutants [35]. 

In addition to nutrient starvation, autophagy can also be induced by leaf senescence, during which nutrients are reallocated from the source leaves to sink organs, such as young leaves or seeds, to maximize plant productivity [36,37,38]. In cereal crops, nitrogen remobilized from senescent leaves can make up 50% to 90% of the nitrogen in seeds [39]. While the expression of a set of *ATG* genes is significantly induced during *Arabidopsis* leaf senescence, the autophagic degradation of chloroplastic proteins via Rubisco-containing bodies (RCBs), or the whole chloroplast, occurs in senescent leaves. Considering that about 75% to 80% of total leaf nitrogen is stored with chloroplasts, this kind of autophagic chloroplast degradation during leaf senescence has been considered as an important mechanism contributing to nitrogen remobilization [40,41,42]. In crops, upregulated expressions of *ATG* genes during nitrogen redistribution-associated leaf senescence are reported in soybean, apple, and barley [40,41,42,43]. In natural senescent maize leaves, the ratio of the lipidated ATG8 to the free ATG8 is found to be markedly increased by senescence, suggesting that a high rate of autophagy occurs during this process [30].

### 2.2. Autophagy-Dependent Recycling and Remobilization of Nitrogen 

The acute response of autophagy to both nutrient deprivation and leaf senescence suggests the important role of autophagy in nutrient recycling and remobilization. Using ^15^N tracing, autophagy-dependent recycling and the remobilization of nitrogen have been first established in *Arabidopsis* at the whole-plant level. By monitoring ^15^N fluxes from *Arabidopsis* rosettes to the seeds and calculating the nitrogen remobilization efficiency (NRE) as the indicator, N remobilization is markedly suppressed in the three autophagy-deficient plants (*atg5-1* mutant, *atg9-2* mutant, and *atg18a* RNAi lines), irrespective of the nutrient conditions, but this defect is more significant under low nitrate conditions [15]. In line with this, larger amounts of ammonium, amino acids, and proteins are accumulated in these *atg* mutants than the wild type [44]. Likewise, nitrogen partitioning studies in the maize *atg12* mutants reveal that autophagy deficiency causes more nitrogen to accumulate in vegetative tissues, such as the stalks and upper leaves, but less nitrogen remobilizes to the seeds during seed fill, which significantly decreases the seed yield [13]. Autophagy blockage in the rice *Osatg7* mutant is found to suppress nitrogen remobilization during the vegetative growth period, which results in a significant decrease in the nitrogen supply from senescent leaves to the newly expanding leaves and further reduces leaf area and tillers. The degradation of soluble proteins in senescing leaves, especially Rubisco, is impaired in the *Osatg7-1* mutant, suggesting that the blockage of the autophagic degradation pathway of chloroplastic proteins accounts for the defects of nitrogen remobilization [28,34]. In addition, it has been reported in recent years that, by overexpressing *ATG* genes, plant tolerance to nitrogen limitation could be improved. *GmATG8c* from soybeans can be dramatically induced by nitrogen starvation, and the constitutive overexpression of *GmATG8c* in soybean calli improves the tolerance to nitrogen deficiency. The heterologous expression of *GmATG8c* in *Arabidopsis* also leads to better performance under nitrogen starvation and an increased yield [32]. In foxtail millet, the expression of *SiATG8a* is highly responsive to nitrogen starvation treatment, and the heterologous expression of *SiATG8a* in *Arabidopsis* and rice can improve plant tolerance to nitrogen limitation stress [33,45]. The overexpression of apple *MdATG8i*, *MdATG3b*, or *MdATG9* in the ‘Orin’ apple callus or *MdATG18a* in apple plants improves the growth performance when nitrogen or carbon supplies are limited, and their ectopic expression in *Arabidopsis* also increases the tolerance of plants to nitrogen or carbon starvation [40,46,47,48]. In rice, the overexpression of *ATG8* family members, including *OsATG8a*, *OsATG8b*, and *OsATG8c*, can significantly increase nitrogen-use efficiency and further improve grain yield, while the *osatg8b* knock-out mutants show reduced nitrogen remobilization and grain yield [49,50,51,52]. All these findings clearly demonstrate that autophagy is essential for nitrogen recycling and remobilization.

### 2.3. Autophagic Recycling and Remobilization of Micronutrients and Sulphur

In addition to nitrogen remobilization, autophagy has been recently established to be involved in the remobilization of iron (Fe) and S from source organs to seeds by using ^57^Fe and ^34^S pulse labeling in *Arabidopsis* [26,53]. The autophagy defect in the *atg5* mutant leads to a drastic reduction in Fe translocation efficiency from vegetative organs to seeds [53], and *^34^*S remobilizations from the rosettes to the seeds are also significantly impaired in the *atg5* mutants, irrespective of sulfur nutrition [26]. Additionally, autophagy is likely to take part in the remobilization of Zn and manganese (Mn) to seeds, because their concentration in the dry remains of *atg* mutants (*atg5-1* and *atg4a atg4b-1*) is higher than the wild-type controls, and their translocation efficiencies to seeds is much lower [53]. However, these results need to be further confirmed using radiotracer labeling experiments. Autophagy is essential for increasing Zn bioavailability under Zn limitation, and autophagy blockage leads to a reduction in the amount of free Zn in *Arabidopsis* plants [25]. However, in maize plants, no obvious impacts of P and S limitations are found on the growth of soil-grown *atg12-1* and *atg12-2* mutants [13]. Whether autophagy participates in the remobilization and recycling of nutrient elements, other than nitrogen, remains unknown in crops. 

### 2.4. The Autophagy-Dependent Remobilization of Carbonhydrates 

Leaf starch, a major integrator in the regulation of plant growth, is synthesized in chloroplasts during the day and hydrolyzed to maltose and glucose during the night to sustain metabolism and growth [54]. Autophagy has been demonstrated to participate in the diurnal degradation of this kind of transitory starch accumulated in the leaves of *N. benthamiana* grown under a long-day photoperiod (16 h light/8 h dark). Small starch granule-like structures (SSGLs), exported from chloroplasts, could be sequestered in autophagosomes and delivered into vacuoles for depletion [55,56]. This autophagy-dependent leaf starch degradation pathway is an ideal supplement to the classical chloroplastic pathway to ensure the timely remobilization of leaf starch and maximum support to the continued nocturnal plant growth during the daylength changes [57]. Indeed, starch accumulation in leaves by dawn was not observed in *Arabidopsis atg* mutants grown under the short-day photoperiod (10 h light/14 h dark) [58]. Whether a similar degradation pathway of leaf starch occurs in crops remains unknown, since little research has been conducted related to the leaf starch metabolism during the diurnal cycle in crop species grown under normal conditions. 

### 2.5. Autophagy-Dependent Lipid Metabolism

Through the multi-omics analysis of the *atg12* mutant and wild-type maize plants, autophagy deficiency strongly alters leaf metabolism, regardless of the nutritional status, especially those with regard to membrane turnover and subsequent lipid breakdown, and the accumulation of secondary metabolites associated with flavonoid biosynthesis. The lower levels of intact phospholipids and the higher levels of complex lipids breakdown products accumulated in the maize *atg12* mutant, such as fatty acids, lysolipids, oxylipins, and glycerolipids, suggesting the importance of autophagy for lipid homeostasis [59,60]. In *Arabidopsis*, basal autophagy has been connected to triacylglycerol (TAG) synthesis, by contributing to fatty acid mobilization from membrane lipids to TAGs, while, upon dark-induced carbon starvation, autophagy is induced to assist TAG hydrolysis by the vacuolar degradation of lipid droplets in a way resembling microlipophagy [53]. More cytoplasmic lipid droplets are indeed observed in the *atg12-1* maize mutant, even under normal growth conditions [60]. In rice, autophagy occurring in postmeiotic tapetum cells promotes the vacuolar degradation of lipid droplets, and the autophagy defect in the *Osatg7* mutant reduces the lipid droplet numbers in the pollen grain and impairs phosphatidylcholine editing and lipid desaturation, which impedes pollen maturation [35]. The autophagy-mediated decomposition of lipid droplets via the microlipophagy-like process has also been described in germinating castor bean seeds to provide energy [61].

## 3. The Role of Autophagy during Development

### 3.1. Vegetative Growth

Beyond the growth retardation induced by nutrient deficiency, reduced growth is also observed in the autophagy-defective plants of *Arabidopsis* (*atg2*, *atg5*, *atg7*, *atg9*, *atg10*, and *ATG18a* RNAi) grown in nutrient-rich soils under a short-day photoperiod (8 h or 10 h of light) [15,19,62], suggesting the role of autophagy for the general vegetative growth of plants. Likewise, this impact of autophagy deficiency on vegetative development is also observed in the rice *Osatg7-1* mutant grown under the 14 h photoperiod, which shows smaller shoots and roots, and reduced leaf area and tillers than the control plants, throughout the growth stage under ample-nutrient conditions [34]. However, this defect of vegetative growth is not apparent in *Arabidopsis atg5* and *atg7* mutants grown under continuous light conditions, as well as maize *atg12* mutants grown under a long-day photoperiod (16 h light/8 h dark), which grow and develop normally under well-fertilized conditions, and are phenotypically undistinguishable from the wild type [13,58]. Thus, the impacts of autophagy deficiency in plants grown under nutrient-rich conditions seem to vary, depending on the photoperiods.

Additionally, another common phenotype for autophagy-defective mutants is accelerated leaf senescence, which reveals a negative regulatory role of autophagy during plant senescence. Similar early senescence is observed in the rice *Osatg7-1* mutant under favorable growth conditions, as described in *Arabidopsis atg* mutants [34]. Excess salicylic acid accumulation was demonstrated to be the major cause of leaf senescence in *Arabidopsis atg* mutants [62,63]. However, no changes in salicylic acid levels, but a consistent increase in another stress hormone, abscisic acid, was detected in the maize *atg12* mutants [59], suggesting the molecular mechanisms underlying the premature senescence in autophagy-defective plants might be different between species. Intriguingly, *Arabidopsis* ATG8 has been reported to promote senescence by interacting with ABS3, a member of multidrug and toxic compound extrusion (MATE) family transporters, but this role of ATG8 is established to be independent of its canonical function [64]. Similar conflicting results showing the positive involvement of ATG18 in senescence were recently reported in maize plants. Near-isogenic lines with lower *ZmATG18b* expressions and higher *ZmGH3.8* expressions show delayed leaf senescence and a good yield performance, while those with higher *ZmATG18b* expressions and lower *ZmGH3.8* expressions show accelerated leaf senescence and a poor yield performance [65]. However, since the direct correlations between autophagy and the senescence phenotypes are not investigated in this study, it remains unknown as to whether autophagy can exert a positive role during senescence. 

The autophagy defect also affects root development, especially under nutrient-depleted conditions. The growth of primary roots in several *Arabidopsis atg* mutants, such as *atg5*, *atg7*, and *atg4a4b*, are demonstrated to be significantly suppressed by nitrogen- or sugar-deprived conditions [10,11,66,67]. Additionally, reduced numbers of total lateral roots and the total length of lateral roots per primary root are observed in nitrogen-starved *atg4a4b-1* seedlings, suggesting a role of autophagy in lateral root development during nutrient limitation. Indeed, autophagy has been connected to the auxin-dependent lateral root development in *Arabidopsis* under phosphate-starved conditions, possibly by the selective depletion of repressors of auxin accumulation, in cooperation with PUB9 [66,68]. The maize *atg12* mutants also showed stunted root growth when fertilized with low nitrogen [13]. The ectopic expression of apple *MdATG9* in *Arabidopsis* can significantly alleviate the negative effects of nitrogen deprivation on the root lengths and the total number of lateral roots [47]. In addition to be induced by nutrient starvation, autophagy has been previously reported to occur constitutively in the root-tip cells of *Arabidopsis*, irrespective of nutrient status, suggesting a general role during root development [67,69,70]. Consistent with this, a reduction in root growth occurs in the rice *Osatg7* mutant, even under ample-nutrient conditions [34]. The knockdown of *OsATG8b* in rice seedlings leads to shorter roots than the wild-type controls and *OsATG8*-overexpressing seedlings after 7-day germination in water [51]. Furthermore, autophagy and its upstream negative regulator, TOR (Target Of Rapamycin) kinase, have been reported to regulate glucose-mediated root meristem activity [71,72]. The TOR pathway has also been recently reported to be involved in adventitious root formation in *Arabidopsis* and potato [73]; however, whether this process is directly autophagy-dependent remains unknown. 

### 3.2. Reproductive Development

Sexual reproduction in higher plants is a pivotal step in generate progenies and thus crucial for crop yield. Although pollen germination defects and male sterility have been previously reported in the *Arabidopsis* mutant lacking *ATG6* [67,74,75], whether these phenotypes are directly caused by autophagy deficiency remains questionable, since all the reported *Arabidopsis atg* mutants, other than *atg6*, are fertile [10,11,12,16,17,18,19,20,21]. Normal pollen development and germination in *Arabidopsis atg2*, *atg5*, and *atg7* mutants are further confirmed in recent research [69]. In addition, the maize *atg12* mutants are also fertile under normal experimental conditions [13]. In rice, however, autophagy is established to be required for male reproductive development. The rice Os*atg7* mutants are sterile and exhibit limited anther dehiscence, defective pollen maturation, and reduced pollen germination [35]. The possible defective autophagy-dependent degradation and programed cell death of the tapetum, which is the supplier of metabolites and nutrients to the developing microspores and pollen grains, might be one cause of male sterility. Alternatively, reduced gibberellin in Os*atg7* mutants can partially explain the pollen maturation defect [70,76]. Similarly, a requirement for autophagy during male reproduction has been recently reported in tobacco. Autophagy-mediated compartmental cytoplasmic deletion is essential for tobacco pollen germination, and the inhibition of autophagy by knocking down key *ATG* genes, including *ATG2*, *ATG5*, and *ATG7*, remarkably prevents pollen germination [69]. Of note, theses contrasting phenotypes associated with male sterility in the autophagy-defective plants of *Arabidopsis*, rice, and tobacco indicate that the contribution of autophagy to male production varies between different plant species. In addition, autophagy has been reported to participate in the programed cell death of florets in wheat, which is caused by the possible nutrient limitation during increased carbohydrate consumption under long-day conditions [77].

Intact autophagy is required for higher seed production. The *Arabidopsis atg* mutants show lower seed production than the wild-type controls under normal conditions [15,78]. Reduced seed yield is also reported in the well-fertilized maize *atg12* mutants [13]. While the overexpression of *ATG5* or *ATG7* in *Arabidopsis* leads to an enhanced seed set [79], the overexpression of *OsATG8a*, *OsATG8b*, and *OsATG8c* in transgenic rice also improves grain yield [49,50,51], further strengthening the importance of autophagy for productivity. In addition to seed production, autophagy has been reported to be connected with seed quality in rice. Seeds harvested from the rice *Osatg7-1* mutant at a low frequency are smaller and exhibit a chalky appearance and lower starch content in the endosperm [80]. Similar poor quality seeds with chalkiness and smaller, loosely packed starch granules are described in rice *OsATG8b*-RNAi plants [51]. Furthermore, recently, it has been found that the expression of *RcATGs* in castor beans is up-regulated during the later stage of seed-coat development [61], and autophagy occurs in developing wheat grains to accomplish the programed degradation of pericarp cells for the regulation of pericarp thickness [81]. These findings suggest a contribution of autophagy to the seed development of crops.

Recent research reports concerning grapes, peppers, and strawberries indicate the involvement of autophagy in fruit ripening. Two waves of increased autophagy flux are detected during strawberry ripening, and autophagy inhibition by either 3-MA (3-methyladenine) treatment or knocking down *ATG5* and *ATG7* dramatically affects strawberry growth and ripening [82]. Additionally, during the fruit ripening of grapes and peppers, increased transcripts related to autophagy are detected in the grape berry skin and pepper fruit [81,83]. 

## 4. The Role of Autophagy in the Responses to Abiotic Stress

In addition to nutrient starvation, investigations into the roles of autophagy during abiotic stress in model plants reveal that autophagy is vital for conferring resistance to heat, chilling, drought, salinity, hypoxia, or oxidative stresses [84,85,86,87,88]. A growing body of research on crops, such as tomato, apple, wheat, pepper, barley, foxtail millet, and pear, also reveals the rapid transcriptional response of autophagy-related genes to abiotic stresses [29,31,33,40,45,46,84,89,90,91,92,93,94,95,96,97,98], suggesting the possible involvements of autophagy in the abiotic stress tolerance of crops. The detailed research findings are reviewed as follows: 

### 4.1. Temperature Stress 

During heat stress, the expression levels of *ATG5*, *ATG7*, and *NBR1* in tomato plants are elevated after 2–4 h of heat stress in the WRKY3-dependent manner, and the numbers of the LysoTracker-stained autophagic structures in the wild-type controls are also found to be significantly increased during the stress. In consistence with the findings in *Arabidopsis*, autophagy inhibition, by the silencing of *ATG5*, *ATG7*, and *NBR1*, compromises tomato heat tolerance, further demonstrating the importance of autophagy in response to heat stress [86,93]. Endogenous melatonin levels in tomatoes are reported to increase in response to heat stress, and exogenous melatonin treatment or endogenous melatonin manipulation by overexpressing *ASMT*, a gene involved in melatonin synthesis, can enhance thermotolerance by increasing the expression of *ATG* genes and the number of autophagic structures [99]. During the response to chilling stress, brassinosteroids in tomatoes act as a positive regulator of NBR1-dependent selective autophagy to contribute to cold tolerance. The signaling element of brassinosteroids, BZR1, is required for the induced expression of *ATG2*, *ATG6*, and *NBR1* by the chilling stress via the direct binding to their promoters, while the silencing of these *ATGs* or *NBR1* genes compromises BR-induced cold tolerance [84]. In wheat, cold treatment induces the expression of *TaATG4a*, *TaATG4b*, *TaATG8a*, *TaATG8g*, and *TaATG8h* and increases autophagosome numbers in the roots of seedlings [96,100]. Similar induced expression of *ATG* genes and increased autophagic structures are observed in peppers undergoing cold stress [29]. In addition, the expression of *HvATG6* in barley and 26 *SiATG* genes in foxtail millet are reported to be up-regulated by low temperatures [31,33]. 

### 4.2. Drought and Salinity Stress

Drought and high salinity are two of the most common environmental stresses encountered by plants, during which autophagy is demonstrated to be essential [87]. Up-regulated expression of *ATG* genes during these stresses are also found in crop species, such as tomato, wheat, pepper, apple, pear, and foxtail millet [29,33,46,89,92,96,97,98]. In tomato plants HsfA1, a critical transcription factor for tolerance to drought stress, can regulate autophagy by positive transcriptional regulation of *ATG10* and *ATG18f*, while the silencing of *ATG10* and *ATG18f* reduces *HsfA1a*-induced drought tolerance in plants overexpressing *HsfA1a* [89]. In another study, mitochondrial alternative oxidase (AOX)-dependent ROS signaling is critical for autophagy induction in tomato plants, in response to ethylene, and to enhance tolerance to drought stress. During ethylene-induced autophagy, ERF5, a typical drought-responsive transcription factor, also contributes to autophagy induction in tomatoes by promoting the expression of *ATG8d* and *ATG18h* via its binding to the promoters of these two genes [101]. *Medicago truncatula* dehydrin MtCAS31 is recently established to promote tolerance to drought stress by selectively mediating the autophagic degradation of the aquaporin MtPIP2;7 [102]. In apples, *MdATG18a* is transcriptionally induced by various abiotic stresses, including drought, and the overexpression of *MdATG18a* in tomato plants or apple plants markedly enhances their tolerance to drought [90]. The transgenic apple plants overexpressing *MdATG8i* show improved tolerance to both salt and drought [103,104]. Additionally, the expressions of *MdATG3a* and *MdATG3b* are induced by drought, salinity, and oxidative stress, and the ectopic expression of these two genes in *Arabidopsis* improves tolerance to osmotic or salinity stress [46]. In wheat, the expression of all *TaATG8s* and autophagic structure numbers are both induced in response to salt and drought stresses [92]. Autophagy inhibition by 3-MA treatment or knocking down *ATG6* accelerates programed cell death (PCD) in wheat seedlings exposed to drought stress, and autophagy inhibition by silencing of *ATG2* or *ATG7* also promotes PCD occurring during salinity stress, which suggest the essential role of autophagy for tolerance of wheat to drought or salt stress [95]. 

### 4.3. Hypoxia or Oxidative Stress

Autophagy in rice plays an important role for survival under oxidative stress, which is inhibited in autophagy-defective mutants lacking *OsATG10b* [105]. Apple autophagy-related genes, *MdATG3a*, *MdATG3b*, *MdATG8i*, and *MdATG18a*, can all be significantly induced by oxidative stress [40,46]. In wheat, autophagy is critical for the clearance of reactive oxidative species (ROS) during hypoxia stress caused by waterlogging, and autophagy induced by hypoxia can inhibit PCD occurring in the root cells of wheat exposed to short-term waterlogging [94,106].

## 5. The Role of Autophagy in Responses to Biotic Stress

In the last decade, research on the interplay between plant autophagy and diverse types of crop pathogens, including viruses, bacteria, fungi, and oomycetes, has boomed, suggesting the critical roles of plant autophagy in defense against pathogen infection. The major advances are summarized in the following part. 

### 5.1. Autophagy during Virus Infection 

Autophagy protects plants against the infection of three geminiviruses, *cotton leaf curl Multan virus* (CLCuMuV), *tomato yellow leaf curl virus* (TYLCV), and *tomato yellow leaf curl China virus* (TYLCCNV), and, as a result, those viruses are higher accumulated and cause more severe viral symptoms in the autophagy-deficient plants. The antiviral effect on CLCuMuV is achieved by targeting βC1, the virulence factor of CLCuMuV, for autophagic degradation via ATG8 [107]. During the infection of *cauliflower mosaic virus* (CaMV), host autophagy is also activated to guide the vacuolar degradation of the viral capsid protein P4 in an NBR1-dependent manner via the direct binding of NBR1 to P4, by which CaMV initial infection is suppressed [108]. Likewise, an autophagy-dependent antiviral response is also reported during infection of RNA virus. *Turnip mosaic virus* (TuMV) infection activates autophagy in plants, which promotes the autophagic degradation of the RNA-dependent RNA polymerase (RdRp) of TuMV, Nib, by Beclin1 to inhibit virus replication [109]. NBR1-mediated selective depletion of the viral RNA-silencing suppressor HCpro in TuMV also contributes to the suppression of viral accumulation [110]. A similar autophagy-dependent antiviral response is also found in plants infected by *Barley stripe mosaic virus* (BSMV), as the silencing of *ATG5* or *ATG7* in plants enhances BSMV accumulation and viral symptoms [111]. The selective autophagic degradation of p3, an RNA-silencing suppressor protein encoded by *Rice stripe virus* (RSV), by a potential cargo receptor P3IP, is established to be important for suppressing RSV infection [112]. In addition, autophagy induced during *cucumber mosaic virus* (CMV) infection can promote the turnover of the major virulence protein and RNA-silencing suppressor 2b to assist the resistance to CMV [113]. In peppers and *N. benthamiana*, expressions of multiple *ATG* genes are up-regulated after the infection of *Pepper mild mottle virus* (PMMoV), and autophagy inhibition significantly increases the accumulation of PMMoV and aggravated systemic symptoms [114]. 

Apart from the antiviral role of autophagy in virus infection, autophagy has also been reported to be manipulated by some viruses as a strategy to counteract plant resistance, which further strengthens the importance of autophagy for plant defense against viruses, from another perspective. Autophagy manipulation by viruses has been discussed in several excellent review papers [115,116,117], which will not be discussed further here.

### 5.2. Autophagy during Fungi and Oomycete Infection

Autophagy is demonstrated to play an important role in the resistance to necrotrophic fungal pathogens. *Arabidopsis* mutants defective in autophagy exhibit an enhanced susceptibility to the *Botrytis cinerea* or *Alternaria brassicicola* [118]. Similar susceptibility is also observed in the *Arabidopsis atg* mutants inoculated with another necrotrophic fungal pathogen, *Sclerotinia sclerotiorum*, missing its effector oxalic acid (OA) [119,120]. In pear leaves, the silencing of *PbrATG8c* decreases the resistance to *Botryosphaeria dothidea* [98]. By contrast, the stable transgenic *Arabidopsis* plants overexpressing *ATG5* or *ATG7* with stimulated autophagic flux exhibit increased resistance to the *Alternaria brassicicola* [79]. In response to the biotrophic powdery mildew pathogen *Golovinomyces cichoracearum*, *Arabidopsis atg* mutants show enhanced disease resistance, indicating a negative role of autophagy in powdery mildew resistance [121]. In wheat, however, the knocking down of *TaATG6s* is reported to weakly compromise the broad-spectrum powdery mildew resistance, and the knocking down of *TaATG8j* also compromises resistance to stripe rust fungus [122,123], indicating a different role of autophagy during the wheat–biotrophic fungi interaction. In bananas, the preliminary results obtained from 3-MA treatment also suggest a role of autophagy in the resistance to the hemibiotrophic fungal pathogen, *Fusarium oxysporum* [124]. 

A positive role of autophagy is also suggested in response to infection of the oomycete *Phytophthora infestans*. Transient overexpression of *Joka2* or *ATG9* enhanced immunity to *P. infestans*, while the silencing of Joka2 resulted in increased disease lesions in leaves [125,126].

### 5.3. Autophagy during Bacterial Infection

The versatile roles of autophagy are reported during the plant–bacteria interaction. When infected with the virulent bacteria pathogen, *Pseudomonas syringae* pv tomato DC3000 (*Pst*), the autophagy-defective plants in *Arabidopsis*, including the *ATG6*-*AS* plants, *atg7*, and *nbr1*mutants, exhibited enhanced disease susceptibility, suggesting a contribution of autophagy to the basal defense response [127,128,129]. However, conflicting phenotypes are reported in the *atg5*, *atg10*, and *atg18a* mutants infected with the same pathogen [120]. Enhanced resistance is also observed in *GmATG2*-silenced plants infected with *Pseudomonas syringae* pv. glycinea (*Psg*) [130]. These contrasting phenotypes might be related to the dual roles of autophagy during the infection of *Pst*, whose effectors can exploit autophagy for proteasome degradation and enhanced virulence, and can also be targeted by NBR1 for autophagic suppression [127]. In cassava, autophagy deficiency leads to increased disease susceptibility to *Xanthomonas axonopodis* pv. manihotis (*Xam*), which causes cassava bacterial blight [131,132]. Autophagy enhancement in *N. benthamiana* by the silencing of the negative regulator of autophagy, cytoplastic glyceraldehyde-3-phosphate dehydrogenases (*GAPC*), significantly suppresses the growth of the *Pseudomonas syringae* pv tabaci and *Pst* [133], while, in *Arabidopsis*, autophagy activation in mutants of *GAPC1* and the chloroplastic isoform, *GAPA1*, also exhibit enhanced disease resistance to both the virulent *Pst* and avirulent *Pst* expressing the effector AvrRpt2 [134]. Similarly, *MeGAPCs*-silenced cassava shows an improved resistance to *Xam* by autophagy induction [135]. Additionally, a positive role of autophagy is reported during the infection of the necrotrophic bacteria *Dickeya dadantii* in *Arabidopsis* [136].

## 6. Potential Approaches of Autophagy Manipulation for Crop Improvement

### 6.1. Genetic Manipulation of ATG Genes

Mounting research reports about autophagy manipulation by overexpressing *ATG* in plants, especially in crops, have recently emerged, which show the significant improvements in one or more agronomic traits associated with growth, seed yield, or tolerance to abiotic or biotic stresses (Table 1). For instance, *ATG5*- or *ATG7*-overexpressing *Arabidopsis* plants exhibit an increased resistance to necrotrophic pathogens and oxidative stress, delayed aging, and enhanced growth, seed set, and seed oil content [79]; the heterologous expression of soybean *ATG8c* in *Arabidopsis* confers tolerance to nitrogen deficiency and increases yield [32]; the overexpression of the autophagy-related gene *SiATG8a* from foxtail millet improves tolerance to both starvation and drought stress in *Arabidopsis*, while the overexpression of it in the rice increases nitrogen starvation tolerance [33,45]; transgenic apple plants overexpressing *MdATG18a* exhibit an enhanced tolerance to drought and nitrogen deficiency, and transgenic apple plants overexpressing *MdATG8i* improves water-use efficiency [48,90]; and transgenic rice overexpressing *OsATG8a*, *OsATG8b*, and *OsATG8c* show increased nitrogen-use efficiency, yield, and improved seed quality [49,50,51,52]. Notably, few detrimental effects are reported in those plants overexpressing *ATGs*, at least on the visible phenotypes of growth and reproduction, which makes the genetic manipulation of *ATG* genes an approach with a significant potential for crop improvement [137]. However, whether those plants overexpressing *ATGs* will show better performance in the field conditions, when exposed to diverse stresses, remains to be seen.

### 6.2. Genetic Manipulation of Autophagy Regulators

In addition to *ATG* genes, genes that can modulate autophagy are also potential targets for crop improvement (Table 1). Diverse autophagy regulators, such as transcription factors or epigenetic modifiers controlling *ATG* expression or ATG activity, or ATG-interacting partners that can regulate the autophagy pathway, are gradually identified. For instance, the overexpression of the transcription factor HsfA1a in tomatoes increases autophagosome formation and improves drought tolerance, while the overexpression of another transcription factor, BZR1, in tomatoes induces autophagy and enhances tolerance to nitrogen starvation and chilling stress [89,139]. The overexpression of MtCAS3, the selective cargo receptor for the aquaporin MtPIP2;7 in *M. truncatula*, can promote tolerance to drought stress [102]. Knocking down of the negative regulator of autophagy, *GAPCs*, in *N. benthamiana* or cassava increases their resistance to virus or bacterial pathogens [133,135]. Beyond those reported in crops, some newly identified regulators of autophagy could become possible targets for autophagy manipulation in crops. The *Arabidopsis* transcription factor, HY5 (elongated hypocotyl 5), is reported to negatively modulate autophagy by interacting with and recruiting HISTONE DEACETYLASE 9 (HDA9) to transcriptionally suppress the expression of *ATG5* and *ATG8e* [142]. *Arabidopsis* COST1, a recently identified negative regulator of autophagy, can negatively regulate drought tolerance. Of note, although the *cost1* mutant shows increased drought tolerance, its growth is markedly decreased [141]. One bZIP transcription factor, TGA9, is shown to upregulate the expression of ATG8 and activate autophagy under both sucrose starvation and osmotic stress conditions [140]. Whether these positive or negative regulators are suitable for crop improvement should be further evaluated comprehensively, not just based on the growth or yield, but also stress tolerance in the field, and the putative side effects should also be clarified. To obtain an ideal candidate that meets all the standards is not easy and, therefore, a larger pool of candidates for modulating autophagy should be constructed in the future.

### 6.3. Pharmacological Regulation

The pharmacological manipulation of autophagy is another possible approach for autophagy modulation. However, the current reported chemical modulators, including the elicitors, such as BTH (a synthetic analog of salicylic acid); ACC (the precursor of ethylene); brassinosteroids; and melatonin, and the inhibitors, such as 3-MA and Wortmannin, are only lab-used and remain untested in the field [63,84,99,101,143]. High-throughput screens for chemical modulators of autophagy will assist us to identify the efficient, stable, specific, and cost-effective molecular candidates suitable for field applications. If achieved, this pharmacological approach will have great advantages over the genetic manipulation in aspects of either the practical benefit or public acceptance [137,143].

## 7. Future Perspectives

Benefitting greatly from the advancement of autophagy research in the model plants, the study of autophagy in crop species has been rapidly expanding, albeit relatively limited and preliminary. For beneficial agricultural applications, as previously discussed, the further identification and characterization of new autophagy regulators, either genetically or pharmacologically, are critical areas for future research. Considering that multiple biotic and abiotic stresses are usually combined in the field, a more systematic and complete evaluation is needed for crops with autophagy modulated in field conditions. 

## Figures and Tables

**Table 1:** Potential targets for autophagy manipulation to improve the agronomic traits.

Genes	Plant Species	Genetic Manipulation ^a^	Phenotypes	Ref
*GmATG8c*	Soybean	OE	Improved tolerance to N starvation in soybean calli and *Arabidopsis* OE lines	[32]
*SiATG8a*	Foxtail millet	OE	Conferring enhanced tolerance to N starvation in *Arabidopsis* and rice OE lines; improved tolerance to drought stress in *Arabidopsis* OE lines	[33,45]
*MdATG8i*	Apple	OE	Enhanced vegetative growth, leaf senescence and tolerance to N and C starvation in *Arabidopsis* OE lines; better tolerance to N/C starvation in apple OE calli; enhanced tolerance to salt and drought in apple OE lines	[40,103,104]
*MdATG3a, MdATG3b*	Apple	OE	*Arabidopsis* OE lines show accelerated growth and bolting, and improved tolerance to mannitol, NaCl, N, and C starvation; apple calli overexpressing MdATG3b improve tolerance to N and C starvation	[46]
*MdATG7b*	Apple	OE	*Arabidopsis* OE lines show accelerated growth and bolting, and improved tolerance to stresses caused by NaCl and N/C starvation	[138]
*MdATG18a*	Apple	OE	enhanced tolerance to drought stress and N depletion in the apple OE lines; enhanced tolerance to drought stress in the tomato OE lines	[48,90]
*MdATG9*	Apple	OE	Transgenic apple calli confer enhanced tolerance to N depletion; *Arabidopsis* OE lines alleviates the negative effects of N deprivation on the root growth	[47]
*OsATG8a*	Rice	OE	Increased numbers of tillers and reduced height; increased panicle numbers and yield; improved nitrogen use efficiency (NRE) under normal conditions	[49]
*OsATG8c*	Rice	OE	Increased yield under normal conditions; improved NRE under normal or N-deficient conditions	[50]
*OsATG8b*	Rice	OE	conferring higher N-recycling efficiency to grains; increased yield under normal conditions	[51]
*ATG5, ATG7*	*Arabidopsis*	OE	Increased resistance to necrotrophic pathogens and oxidative stress, delayed aging and enhanced growth, seed set, and seed oil content	[79]
*ASMT*	Tomato	OE	Enhanced autophagy and thermotolerance	[99]
*BZR1*	Tomato	OE	Enhanced autophagy and tolerance to chilling stress and N starvation	[84,139]
*HsfA1*	Tomato	OE	Enhanced autophagy and tolerance to drought stress	[89]
*AOX*	Tomato	OE	Enhanced autophagosome formation and ethylene-mediated drought tolerance	[101]
*MtCAS31*	*Medicago truncatula*	OE	Improving drought tolerance by mediating selective autophagic degradation of the aquaporin MtPIP2;7	[102]
*TGA9*	*Arabidopsis*	OE	Increased autophagy under sucrose starvation and osmotic stress; enhanced tolerance to C starvation	[140]
*COST1*	*Arabidopsis*	KO	Increased drought tolerance but decreased growth	[141]
*GAPCs*	*Nicotiana benthamiana*	VIGS	Enhanced resistance to the incompatible pathogens tobacco mosaic virus and *Pst*, as well as compatible pathogen *Pseudomonas syringae* pv *tabaci*	[133]
*GAPC1, GAPA1*	*Arabidopsis*	KO	Enhanced resistance to both the virulent *Pst* and avirulent *Pst* expressing the effector AvrRpt2	[134]
*MeGAPCs*	Cassava	VIGS	to *Xanthomonas axonopodis* pv *manihotis* (*Xam*)	[135]
*HY5*	*Arabidopsis*	KO	Enhanced autophagy and improved tolerance to N/C starvation	[142]

^a^ Genetic manipulation approaches listed in the table including: overexpression (OE), knock-out (KO) and virus-induced gene silencing (VIGS).
